# How does bitterness perception correlate with the intention to use traditional Chinese medicine? An analysis of double-edged sword effects based on the stimuli–organism–response framework

**DOI:** 10.3389/fpubh.2025.1723985

**Published:** 2026-01-14

**Authors:** Guoqing Wang, Mengjing Hua, Xuefeng Xie, Yuanyuan Zhang

**Affiliations:** 1 School of Pharmacy, Anhui Medical University, Hefei, China; 2 Inflammation and Immune Mediated Diseases Laboratory of Anhui Province, Anhui Medical University, Hefei, China; 3 Anhui Provincial Drug Regulatory Scientific Research Center, Anhui Medical University, Hefei, China; 4 Key Laboratory of Public Health Social Governance, Philosophy and Social Sciences of Anhui Province, Anhui Medical University, Hefei, China

**Keywords:** traditional Chinese medicine, TCM bitterness perception, perceived TCM usefulness, perceived TCM uncertainty, intention, TCM cultural identity

## Abstract

**Background:**

This study investigates the relationship between TCM bitterness perception and individuals’ intention to use traditional Chinese medicine (TCM), employing the ‘stimuli–organism–response’ (SOR) framework. The study considers bitterness perception of TCM as stimuli that correlate with two organisms, that are perceived TCM usefulness and perceived TCM uncertainty toward intention to use TCM (response).

**Methods:**

In total, 467 responses are collected from a purposive sample of medical university affiliates with fundamental TCM knowledge using a survey method, and structural equation modeling and ordinary least square regression are applied to provide empirical results.

**Results:**

The result indicates that TCM bitterness perception is positively related to perceived TCM usefulness (perceived TCM uncertainty), through which exerts positive (negative) indirect effect on intention to use TCM. Moreover, high levels of TCM strengthens (attenuates) the relationship between TCM bitterness perception and perceived TCM usefulness (uncertainty), results in more positive indirect effect of TCM bitterness perception on intention to use TCM.

**Conclusion:**

The double-edged sword effects of bitterness perception arise from an asymmetric activation of perceived TCM usefulness and perceived TCM uncertainty at the cognitive stage, which is subsequently counterbalanced through differential transmission strengths, resulting in relatively equal opposing forces on the intention to use TCM. Individuals’ TCM cultural identity plays a contingent role that individuals will make more bitterness-usefulness association and less bitterness-uncertainty association when they hold higher degree of TCM cultural identity, relating to more intention to use TCM. Thus, the results of this study encourage TCM promotion actors leverage the double-edged sword effects of TCM bitterness and the deep power of TCM culture in transforming simple bitterness sensory judgment and sublimates into cultural consciousness. However, due to the specific TCM-literate sample, findings regarding the ‘bitterness-usefulness’ association may not generalize to the general public, where such cultural bridging is weak. And cross-sectional data only demonstrate associations within the tested model.

## 1 Introduction

Traditional Chinese medicine (TCM) is the inheritance of medical science that has lasted for thousands of years (1). The old proverb that “better drugs taste bitter” metaphorically encapsulates that Chinese people searching for therapeutic herbs based on the tastes in daily life practices. For example, TCM theory holds that bitter herbs have the effects of clearing away heat and dampness (such as coptis root) (2). Recent scientific research has confirmed that bitter substances such as alkaloids (berberine) and glycosides (baicalin) do have significant pharmacological activity (3). Given that bitter substances/herbs used in traditional Chinese medicine have a wide range of pharmacological effects, TCM therapeutic formulas composed of such substances/herbs can be used to treat a variety of diseases (4).

However, a central challenge of accepting and adhering to TCM therapy for individuals is a “matter of taste,” with bitter taste a primary culprit. It has been reported that increasingly bitter-tasting medications can lead to a significant decrease in medication compliance (5). The underlying reason is that bitter taste is thought to have evolved to deter the ingestion of toxic substances (6) and may arouse individuals’ innate aversion to use TCM. Despite understanding the pharmacological basis of bitter-tasting compounds and the general compliance challenges they pose, a critical unknown remains in the behavioral literature: the precise psychological pathway through which the subjective perception of bitterness translates into an intention to use TCM. Specifically, it is unclear whether this single sensory stimulus triggers a singular negative reaction or simultaneously activates competing cognitive appraisals—such as interpreting bitterness as a signal of therapeutic potency versus a warning of potential harm—and how these appraisals interact to shape final decisions.

Additionally, the theory of TCM contains rich cultural concepts such as ‘balance of yin and yang’ (7), ‘five elements’ (8), ‘qi’ (9), and so on. Scholars have reached a consensus that TCM culture is deeply rooted in traditional Chinese culture (10–12) whose philosophical system, thinking paradigm and value concepts will subtly influence people’s decision-making. In view of the subtle influence of TCM culture on people’s behavior, this study assumes whether people’s attribution of the bitterness of TCM is contingent on their identification with TCM culture.

Guided by the SOR model, this paper aims to integrate and reconcile the above arguments regarding the confounding effects of bitterness perception of TCM on people’s intention to use TCM. The SOR model believes that individuals, under the stimulation of various external factors (stimuli), make corresponding behavioral changes (response) by judging their internal psychological activities (organisms) (13). Multiple studies based on the SOR model have been conducted in health settings, including adoption of technology protocols for pandemic control (14), mobile health service (15), medicine delivery application (16), online pharmacy services (17). The SOR model is a suitable framework for our research since bitter substances stimulating taste receptors which producing signals relayed to the brain and leading to behavioral outcomes (5). It uniquely investigates bitterness as a stimulus that concurrently influences two distinct and opposing organismic states—perceived TCM usefulness and perceived TCM uncertainty—which in turn exert countervailing forces on the intention to use TCM. Furthermore, it examines TCM cultural identity as a key moderator of the initial stimulus-organism link, a nuanced interaction previously unexplored in this context.

Clarifying this double-edged effect of bitterness taste on the intention to use TCM is not merely of academic interest but has practical urgency. As global interest in TCM grows, effectively promoting its adoption requires moving beyond simple narratives. Understanding whether, when, and for whom bitterness acts as a deterrent or as a culturally coded efficacy signal is necessary for developing targeted, evidence-based communication and intervention strategies that can mitigate aversion and leverage cultural assets to improve public health outcomes.

## 2 Materials and methods

### 2.1 Study design and recruitment

The research hypotheses were empirically tested based on an online self-reported survey using the ‘Questionnaire Star’ platform (https://www.wjx.cn/). It launched as a convenience sampling and finally broadened a snowball sampling in order to recruit more participants. Participants were encouraged to share the link with their family members, friends and colleagues.

As an incentive for increasing the response rate, we set up incentives for participants to win gift cards with a nominal value of 10 CNY. Questionnaires must be completed conscientiously, and incentives will not be issued for invalid responses.

Data collection started on March 25, 2025 and ended on April 28, 2025. We took several measures to mitigate common method bias including (a) ensuring respondent anonymity and clearly stating in the instructions that data would be used solely for academic purposes to reduce social desirability bias, (b) incorporating reverse-coded items within some scales to disrupt response patterns and automatic acquiescence, and (c) inter-mixing items measuring independent and dependent variables throughout the questionnaire to reduce the salience of conceptual relationships for respondents.

Questionnaires completed in less than 2 min or more than 20 min were considered invalid, given that the survey included approximately 27 items, with a reasonable completion time estimated at 5–15 min. Inclusion criteria were as follows: (1) being currently affiliated with a medical university in Anhui Province, China (including both students and faculty members) with fundamental knowledge of TCM. At the beginning of the questionnaire, a screening question was included: “Have you studied relevant courses or otherwise acquired substantial knowledge of TCM?” Participants who answered “yes” proceeded to the main survey, whereas those who answered “no” were exited from the questionnaire. (2) possessing digital accessibility through internet-enabled devices (smartphones or computers); and (3) demonstrating Chinese literacy sufficient to comprehend the questionnaire content; (4).

15–20 observations are proper for each studied variable (18), the expected sample size of our study should be around 440 (totaling 22 items) respondents. Out of the 531 questionnaires distributed to students and faculties, 467 valid ones were kept, with a response rate of 87.95%. The analysis confirmed that our target sample size of approximately 460 respondents was sufficient to detect the hypothesized small-to-moderate effects with adequate power.

To ensure the linguistic validity and cultural accuracy of the questionnaire, we implemented three quality control protocols. First, a standardized back-translation procedure was conducted by bilingual experts to verify conceptual equivalence between the original and translated items. The two bilingual experts held doctoral degrees in pharmacy or public health and were fluent academic translators with prior experience in cross-cultural instrument adaptation. Second, a pilot validation study (N = 25) was administered prior to formal data collection to assess item comprehensibility and identify potential ambiguities, with all pilot data excluded from formal analyses. Based on the pilot feedback, all variables demonstrated good reliability and validity, except for several items measuring the perceived TCM usefulness which exhibited CITC values below 0.5 and did not meet the retention criteria; these items were therefore removed. Third, we employed ethical persuasion strategies by explicitly emphasizing the anonymous nature of the survey and implementing strict confidentiality protocols regarding data usage, thereby reducing respondents’ suspicion or hesitation to fill out the questionnaire. At last, it should be noted that the cross-sectional design identifies associations between constructs and does not establish causal direction.

### 2.2 Theoretical framework

In this study, TCM bitterness perception is operationalized as the Stimulus (S); perceived TCM usefulness and perceived TCM uncertainty are conceptualized as the cognitive Organismic states (O); and intention to use TCM is the ultimate Response (R). The SOR framework was selected over alternative models (e.g., TAM, TPB) for its direct applicability to sensory-driven decision processes. The SOR paradigm functions when environmental factors (stimulus) influence an individual’s inner state such as affective and cognitive systems (organism), which ultimately leads to his or her behavioral changes (response) (13). This is distinct from TAM/TPB, which typically start with pre-formed beliefs or social perceptions (51).

The SOR framework was selected as the theoretical foundation of this study not only because it demonstrates exceptional adaptability in incorporating diverse stimulus variables across behavioral contexts but also elucidates the latent mechanisms underlying unobservable psychological processes through a multidimensional continuum of internal states spanning affective and cognitive. This paradigm uniquely bridges external stimuli with behavioral outcomes while operationalizing the mediation of covert psychological processes, thereby serving as an optimal framework for investigating complex human decision-making pathways. This investigation operationalizes TCM bitterness perception as a gustatory stimulus within the SOR paradigm, activating two distinct organismic states: perceived TCM usefulness and perceived TCM uncertainty. This operationalization aligns with research demonstrating that primary sensory attributes, particularly taste, serve as potent heuristic cues that directly inform risk–benefit assessments and behavioral choices in health contexts (6). These mediating constructs embody the critical organism component, translating gustatory stimulus into behavioral responses.

On the one hand, perceived usefulness has been proved among many empirical tests to be a significant antecedents of usage intention (19). The perceived usefulness of TCM was defined as individuals’ cognitive assessment regarding the extent to which participants believed TCM utilization would positively influence their health outcomes (20). On the other hand, bitter taste is thought to have evolved as a deterrent against ingesting toxic substances (6) and may arouse people’s uncertainty perception of TCM. They represent competing appraisals—a positive assessment of usefulness versus a negative assessment of uncertainty—that are simultaneously activated by the same stimulus, thereby creating the proposed ‘double-edged’ internal conflict.

**Figure 1:**
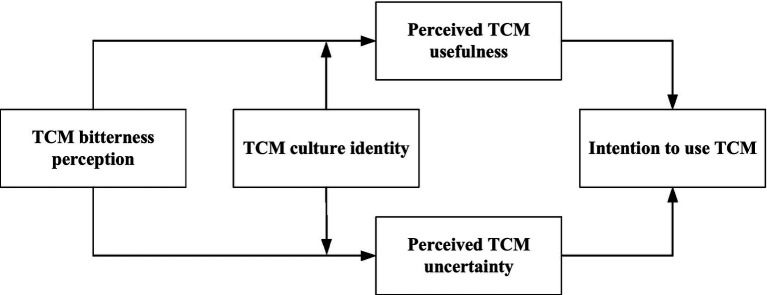
Conceptual framework of the study.

Additionally, in view of the subtle influence of TCM culture on people’s behavior, this investigation advances the SOR paradigm through integration of TCM cultural identity as a critical moderating variable. A stronger cultural identity is expected to enhance the learned bitterness-usefulness association while attenuating the innate bitterness-uncertainty association, thereby rebalancing the competing cognitive appraisals triggered by the stimulus. Figure 1 illustrates the conceptual framework of this study. Guided by the adapted SOR model, we formulate the following empirically testable hypotheses:

*H1:* TCM bitterness perception is positively associated with perceived TCM usefulness.

*H2:* TCM bitterness perception is indirectly associated with intention to use TCM through its positive association with perceived TCM usefulness.

*H3:* TCM bitterness perception is positively associated with perceived TCM uncertainty.

*H4:* TCM bitterness perception is indirectly associated with intention to use TCM through its positive association with perceived TCM uncertainty.

*H5:* TCM cultural identity moderates the positive association between TCM bitterness perception and perceived TCM usefulness. This association is stronger among individuals with high levels of TCM cultural identity compared to those with low levels.

*H6:* The strength of the positive indirect association between TCM bitterness perception and intention to use TCM (via perceived TCM usefulness) depends on TCM cultural identity, such that this indirect association becomes stronger as TCM cultural identity increases.

*H7:* TCM cultural identity moderates the positive association between TCM bitterness perception and perceived TCM uncertainty. This association is weaker among individuals with high levels of TCM cultural identity compared to those with low levels.

*H8:* The strength of the negative indirect association between TCM bitterness perception and intention to use TCM (via perceived TCM uncertainty) depends on TCM cultural identity, such that this indirect association becomes weaker as TCM cultural identity increases.

### 2.3 Questionnaire development

We preliminarily measured the constructs in this study by reviewing related literature. To ensure the questionnaires reliability and validity, adjustments were made to the wording of certain items to align with TCM’s context by resorting to five experts engaged in TCM education, research, or clinical practice. Their feedback mainly focused on clarifying ambiguous expressions to improve precision and removing redundant items to prevent respondent fatigue. During the pilot testing phase, 25 students with pharmacy-related backgrounds completed the questionnaire and provided feedback to further refine the questionnaire.

The validated questionnaire comprised two modules. Module I focused on demographic profiling including gender, age, education, TCM experience, and self-reported health status. Module II operationalized the SOR framework through a theory-driven instrument development protocol, containing 22 validated items across 5 theoretically anchored constructs. Among them, 3 items were used to measure perceived TCM usefulness (20), 4 items were applied to assess the perceived TCM bitterness (21, 22), 5 items were operationalized to present perceived TCM uncertainty (22), TCM cultural identity (21), and intention to use TCM (20, 23). Table 1 documents the complete item pool. All constructs employed 5-point Likert-type scales (1 = strongly disagree; 5 = strongly agree).

**Table 1:** The questionnaire based on the theory of SOR.

Construct	Items	Source
TCM cultural identity (CI)	CI1: TCM is a national quintessence and a treasure of China.	Oladokun et al. (21)
	CI2: TCM treats both symptoms and causes.	
	CI3: It is necessary to set up a TCM major in colleges and universities.	
	CI4: The government should pay more attention to the inheritance and development of TCM culture.	
	CI5: If I am sick, I will not exclude TCM treatment.	
Perceived TCM usefulness (PU)	PU1: I think TCM is helpful in improving my health.	Xia et al. (20)
	PU2: I think TCM plays a fundamental role in daily health care.	
	PU3: I think TCM plays a fundamental role in the treatment of diseases.	
Perceived TCM uncertainty (PUN)	PUN1: I’m concerned about the side effects of TCM.	Eskine et al. (22)
	PUN2: It takes long time to accept TCM treatment.	
	PUN3: It takes long time to understand TCM treatment.	
	PUN4: I’m concerned about the cost-effectiveness of TCM.	
	PUN5: I’m concerned about the efficacy of TCM.	
Perceived TCM bitterness (Bitter)	Bitter 1: I’m sick of the bitter taste of TCM.	
	Bitter 2: The bitter taste is lingering in the mouth after taking TCM.	Oladokun et al. (21) and Eskine et al. (22)
	Bitter 3: The bitter taste of TCM is astringent.	
	Bitter 4: The bitter taste of TCM makes me feel disgusting.	
Intention to use TCM (TCMI)	TCMI1: I prefer TCM for healthcare.	Xia et al. (20) and Ng et al. (23)
	TCMI 2: I will choose TCM to treat diseases.	
	TCMI 3: I would like to use TCM in the future.	
	TCMI 4: I will recommend relatives, friends and colleagues to choose TCM.	
	TCMI 5: I’m willingness to pay for TCM products.	

### 2.4 Data analysis

The analytical sequence was designed to leverage the strengths of different methods. First, confirmatory factor analysis (CFA) and structural equation modeling (SEM) in Amos were used to validate the measurement model and test the direct and indirect relationships among latent variables (H1-H4), as SEM optimally handles measurement error and model complexity. Subsequently, to test the continuous moderation hypotheses (H5, H7) with maximum clarity and statistical power, we employed hierarchical ordinary least squares (OLS) regression in STATA using composite scores. This approach avoids the complexity and potential convergence issues associated with latent interaction modeling in SEM, while providing a robust and interpretable test of the interaction effects. The conditional indirect effects (H6, H8) were then calculated using the regression-based path coefficients within a bootstrapping framework. All analyses were executed by using SPSS (v25.0, IBM Corp., Armonk, NY), Amos (v26.0, IBM Corp.), and STATA/BE (v17.0, StataCorp LLC, College Station, TX).

## 3 Results

### 3.1 Demographical characteristics of participants

Out of the 531 questionnaires distributed to students and faculties, 467 valid ones were kept, with a response rate of 87.95%. More than half of the participants were female (54.60%). Respondents were largely above 20 years old (74.30%), had educational level of bachelor or above (80.30%), and had TCM using experience (61.24%). The self-report health status of respondents was mainly normal (43.68%) and good (37.26%). The detailed demographical characteristics of the final sample are described in Table 2. More than half of the participants were female (54.60%), most of the age were 20 years old or above (74.30%), educated at the bachelor level or above (80.30%). Respondents (61.24%) had TCM using experience, and nearly half of them (43.68%) were in normal health status.

**Table 2:** Descriptive statistics of demographic characteristics.

Variable	Categories	Percentage
Gender	Female	54.60%
	Male	45.40%
Age	<20	25.70%
	> = 20	74.30%
Education	Below bachelor	19.70%
	Bachelor level or above	80.30%
I have TCM using experience	Yes	61.24%
	No	38.76%
Health status	Very bad	0.21%
	Bad	5.14%
	Normal	43.68%
	Good	37.26%
	Very good	13.70%

### 3.2 Measurement validity and descriptive statistics

#### 3.2.1 Reliability assessment

Internal consistency reliability was first assessed using Cronbach’s alpha, acknowledging its known sensitivity to the number of items and its assumption of tau-equivalence. As presented in Table 3, the Cronbach’s alpha value for the five measured variables ranged between 0.837 and 0.925, substantially exceeding the conventional reliability threshold of 0.700 (24). Composite reliability (CR), which makes fewer restrictive assumptions and is derived from the factor loadings, was also computed as a more robust indicator. Composite reliability indices demonstrated strong internal consistency (CR = 0.844–0.928), surpassing the recommended 0.70 benchmark (24).

**Table 3:** Test of reliability and convergent validity.

Constructs	Items	Factor loading	Cronbach’s alpha	CR	AVE
Bitter	B1	0.869	0.837	0.881	0.652
	B2	0.757			
	B3	0.887			
	B4	0.844			
CI	CI1	0.866	0.925	0.928	0.721
	CI2	0.803			
	CI3	0.844			
	CI	0.888			
	CI5	0.786			
PU	PU1	0.791	0.917	0.844	0.644
	PU2	0.809			
	PU3	0.807			
PUN	PUN1	0.819	0.871	0.896	0.633
	PUN2	0.852			
	PUN3	0.759			
	PUN4	0.785			
	PUN5	0.822			
TCMI	I1	0.825	0.913	0.918	0.692
	I2	0.875			
	I3	0.878			
	I4	0.781			
	I5	0.852			

#### 3.2.2 Convergent validity

Convergent validity was rigorously evaluated using Fornell and Larcker (24) tripartite validation framework: (1) All factor loadings in Table 3 exceeded the threshold of 0.700, (2) composite reliability indices demonstrated strong internal consistency (CR = 0.844–0.928), surpassing the recommended 0.70 benchmark, and (3) average variance extracted estimates (AVE = 0.580–0.710) consistently exceeded the 0.500 criterion, indicating that over 50% of variance in latent constructs was explained by their respective indicators rather than measurement error.

#### 3.2.3 Discriminant validity

Discriminant validity meets the statistical criterion if the square root of AVE for each construct exceeds its bivariate correlation coefficients with other latent variables. As demonstrated in Table 4, the bolded diagonal elements (square root of each AVE values) surpassed all off-diagonal inter-construct correlations. This pattern confirms that each latent construct shares more variance with its designated indicators than with conceptually distinct measures, thereby satisfying the fundamental requirement for empirical distinctiveness in measurement models. Discriminant validity was further confirmed using the Heterotrait-Monotrait ratio (HTMT) of correlations (25). All HTMT values were below the recommended threshold of 0.85, supporting the empirical distinctiveness of the constructs.

**Table 4:** Descriptive statistics, correlations of measurements and validity.

Constructs	Mean	S.D.	Bitterness	CI	PU	PUN	TCMI
Bitter	3.240	0.753	**0.807**				
CI	4.458	0.625	0.072	**0.849**			
PU	4.009	0.712	0.114**	0.564***	**0.802**		
PUN	3.116	0.795	0.312***	−0.136**	−0.154***	**0.795**	
TCMI	3.385	0.772	0.035	0.326***	0.501***	−0.219***	**0.832**

The square root of average variance extracted in bold on diagonal, and other values represent Pearson correlation. CI denotes TCM culture identity; Bitter denotes perceived TCM bitterness; PU denotes perceived TCM usefulness; PUN denoted perceived TCM uncertainty; TCMI denotes intention to use TCM. *p < 0.05; **p < 0.01; ***p < 0.001.

#### 3.2.4 Assessment of multicollinearity

Table 4 presents descriptive statistics and intercorrelations for all study variables excluding demographic controls. The observed correlation coefficients and variance inflation factors (VIFs) across models all VIFs < 1.610, substantially below the commonly recommended threshold of 5 indicate no evidence of problematic multicollinearity in our analyses (26).

### 3.3 Common method bias

The study employed two approaches to assess common method bias. First, a Harman’s single-factor test was conducted (27). The analysis extracted five factors with eigenvalues greater than 1, which together accounted for 74.13% of the total variance. The first factor explained 30.85% of the variance, which is less than half of the total, providing an initial indication that common method bias was not a severe concern in this dataset. Second, a more rigorous test was performed by comparing a baseline measurement model with a model controlling for the effects of an unmeasured latent methods factor (ULMC) (27). As shown in Table 5, the differences in key fit indices between the baseline model and the common method factor model were negligible (∆CFI/∆GFI/∆IFI/∆TLI < 0.01; ∆SRMR/∆RMSEA < 0.05). This confirms that the common method factor did not substantially improve model fit, indicating the absence of significant common method bias in our data.

**Table 5:** Common method bias test based on ULMC.

Model	χ^2^/df	RMSEA	RMR	CFI	GFI	IFI	TLI
Baseline	2.319	0.053	0.037	0.961	0.918	0.961	0.955
ULMC	2.077	0.048	0.027	0.972	0.933	0.972	0.963
Difference	0.242	0.005	0.01	−0.011	−0.015	−0.011	−0.008

χ^2^/df, relative chi-square and degrees of freedom; RMSEA, root mean square error of approximation; RMR, root mean square residual; CFI, comparative fit index; GFI, goodness-of-fit index; IFI, incremental fit index; TLI, ‌Tucker-Lewis Index‌.

### 3.4 Goodness-of-fit statistics

As delineated in Table 6, although the Comparative Fit Index (CFI = 0.898) is slightly below the strict 0.90 threshold, the collective pattern of other fit indices supports an acceptable model fit (RMSEA = 0.067 < 0.08; RMR = 0.019 < 0.05; GFI/AGFI/IFI > 0.90). It is recognized in methodological literature that for models incorporating complex effects, the CFI can be slightly attenuated while the model retains substantive explanatory value (28). The consistent results across other indices suggest a reasonable fit between the hypothesized model and the observed data.

**Table 6:** Overall fitness evaluation standard and fitting evaluation result of structural equation model.

Index category	Index name	Actual fit	Compared with evaluation criteria	Result
	χ^2^(22)	68.484		
Absolute fit index	χ^2^/df	3.113	< 5.00	Ideal
	RMSEA	0.067	< 0.080	Ideal
	RMR	0.019	< 0.050	Ideal
	GFI	0.972	> 0.900	Ideal
	AGFI	0.930	> 0.900	Ideal
Incremental fitness index	CFI	0.898	> 0.900	Acceptable
	IFI	0.903	> 0.900	Ideal

χ^2^/df, relative chi-square and degrees of freedom; RMSEA, root mean square error of approximation; RMR, root mean square residual; GF, goodness-of-fit index; AGFI, adjusted goodness-of-fit index; CFI, comparative fit index; IFI, incremental fit index.

### 3.5 Hypotheses testing

**Figure 2:**
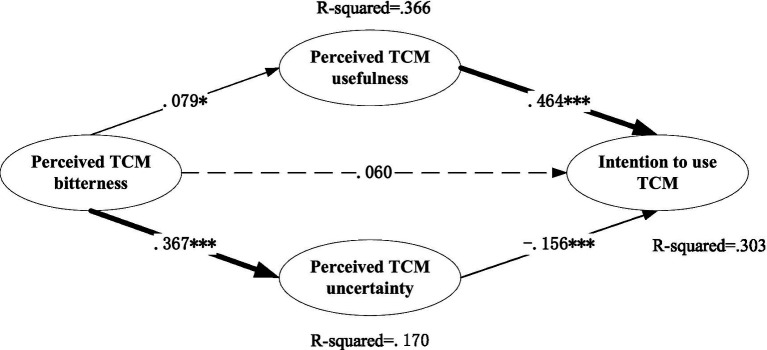
Structural equation model on intention to use TCM based on the theory of SOR. Standardized path coefficients were presented; *p < 0.05; **p < 0.01;***p < 0.001.

We employed SEM to examine Hypotheses 1 through 4, evaluating hypothesized relationships through a comprehensive analysis of measurement and structural models. As shown in Figure 2, TCM bitterness perception has significant positive effects on the perceived TCM usefulness (*β* = 0.079, *p* < 0.05, SE = 0.036) and perceived TCM uncertainty (*β* = 0.367, *p* < 0.001, SE = 0.045), supporting H1 and H3. For the pathway linking TCM bitterness perception to perceived usefulness of TCM (*β* = 0.079, *p* < 0.05), we computed local effect size f^2^ using the R^2^ incremental method (29). After controlling for five covariates, the baseline model yielded R₁^2^ = 0.009, while the full model including bitterness perception produced R₂^2^ = 0.022. This results in f^2^ = (R₂^2^ – R₁^2^)/(1 – R₂^2^) ≈ 0.013, which falls below the conventional threshold of 0.02 for a small effect according to Cohen’s criteria. The modest magnitude of this association is conceptually plausible in behavioral studies of TCM, where usage intention is shaped by multiple intertwined factors—such as cultural identity, prior experience, and outcome expectations—rather than by a single sensory cue like bitterness.

Further, perceived TCM usefulness and perceived TCM uncertainty have significantly positive (*β* = 0.464, *p* < 0.001, SE = 0.051) and negative (*β* = −0.156, *p* < 0.001, SE = 0.041) influence on intention to use TCM, respectively. The indirect effects were estimated using 5,000 bootstrap resamples (30). Results revealed two distinct mediation pathways: First, perceived TCM bitterness exerted a significant positive indirect effect on intention to use TCM through enhanced perceived usefulness (*β* = 0.037, *p* = 0.019), with the 95% bias-corrected confidence interval excluding zero [0.007, 0.072], thereby confirming Hypothesis 2. Conversely, a significant negative indirect effect emerged through increased perceived uncertainty (*β* = −0.057, *p* = 0.001), supported by the non-zero-crossing confidence interval [−0.099, −0.025], which strongly validates suppression mechanism proposed by Hypothesis 4.

Above analysis reveals a difference in the strength of bitterness perception’s influence on the two mediators: its direct positive effect on perceived uncertainty (*β* = 0.367) is significantly stronger than its effect on perceived usefulness (*β* = 0.079), with the former being approximately 4.5 times the latter. This indicates that bitterness, as a strong sensory stimulus, exhibits a notably stronger innate link to ‘potential risk’ than its culturally acquired link to ‘therapeutic efficacy,’ reflecting asymmetric activation. Meanwhile, usefulness (path coefficient = 0.464) and uncertainty (path coefficient = −0.156) differ in their driving strength on behavioral intention, leading to differential amplification or attenuation along the two pathways. As denoted in Table 7, the total indirect effect of bitterness on intention is decomposed as follows: the positive indirect effect via perceived usefulness is 0.079 × 0.464 = 0.037, while the negative indirect effect via perceived uncertainty is 0.367 × (−0.156) = −0.057. The absolute ratio of these two indirect effects is approximately 1:1.5, indicating that, at the endpoint of the complete ‘stimulus–organism–response’ chain, the two opposing cognitive forces activated by bitterness perception exert a relatively balanced influence on behavioral intention, thereby forming the empirical basis of its ‘double-edged sword’ effect. The total effect of perceived TCM bitterness on the intention to use TCM can be calculated as the sum of direct effect and indirect effects. Given the insignificant direct effect and the countervailing indirect effects, the total effect is positive and insignificant as well (detailed in Table 7).

**Table 7:** Model effects analysis.

Model effects			Confidence interval		
	Effect size	Boot SE	Lower	Upper	Percentage
Total effect	0.04	0.05	−0.006	0.138	100%
Direct effect	0.06	0.046	−0.028	0.153	38.96%
Indirect effect: *Bitterness-PU-TCMI*	0.037*	0.016	0.007	0.072	24.03%
Indirect effect: *Bitterness-PUN-TCMI*	−0.057***	0.019	−0.099	−0.025	37.01%

*p < 0.05; **p < 0.01; ***p < 0.001.

**Figure 3:**
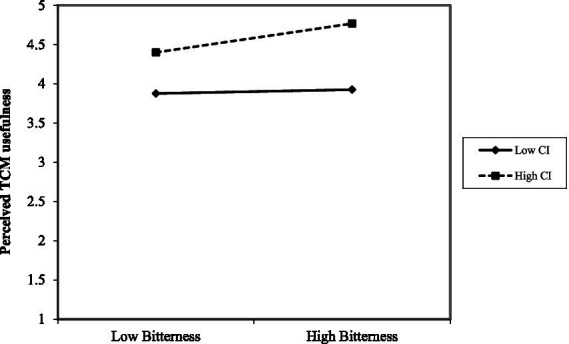
The moderating effect of TCM cultural identity on the relationship between TCM bitterness perception and perceived TCM usefulness. CI denotes TCM culture identity; Bitterness denotes perceived TCM bitterness.

**Figure 4:**
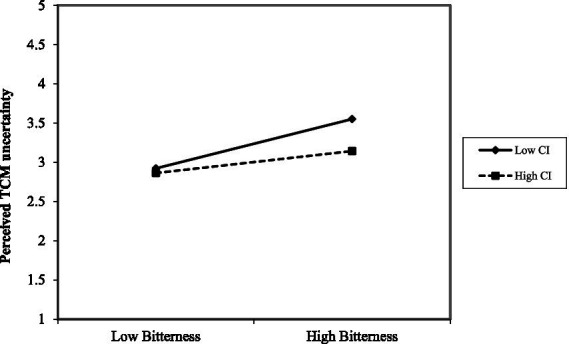
The moderating effect of TCM cultural identity on the relationship between TCM bitterness perception and perceived TCM uncertainty. CI denotes TCM culture identity; Bitterness denotes perceived TCM bitterness.

To test the moderating hypotheses (H5 and H7), OLS regression analyses were conducted. As shown in Table 8 (Models 3 and 6), the interaction terms between centered perceived TCM bitterness and TCM cultural identity demonstrated statistically significant effects, providing evidence for the moderating hypotheses. In Figure 3, for individuals with high TCM cultural identity (+1 SD), the relationship between bitterness and usefulness was positive and significant (*β* = 0.244, *p* = 0.027), whereas for those with low cultural identity (−1 SD), it was non-significant (*β* = 0.034, *p* = 0.448). By contrast, Figure 4 indicates that individuals with high TCM cultural identity (+1 SD), the relationship between bitterness and uncertainty of individuals with high TCM cultural identity (+1 SD, *β* = 0.185, *p* = 0.093) was less positive and significant than individuals with high TCM cultural identity (−1 SD, *β* = 0.418, *p* = 0.000).

**Table 8:** Regression results of the moderating role of TCM culture identity.

Variables	Model 1	Model 2	Model 3	Model 4	Model 5	Model 6
	PU	PU	PU	PUN	PUN	PUN
Gender	0.062	0.077	0.073	0.031	0.1	0.105
	(0.055)	(0.056)	(0.055)	(0.074)	(0.070)	(0.070)
Age	−0.043	−0.039	−0.027	0.114	0.132*	0.119
	(0.063)	(0.063)	(0.063)	(0.084)	(0.079)	(0.079)
Education	−0.084	−0.094	−0.090	0.077	0.031	0.026
	(0.070)	(0.069)	(0.069)	(0.093)	(0.087)	(0.087)
Health status	0.048	0.048	0.043	−0.106**	−0.110**	−0.104**
	(0.034)	(0.034)	(0.034)	(0.046)	(0.043)	(0.043)
TCM experience	0.004	0.024	0.005	0.060	0.152**	0.173**
	(0.057)	(0.057)	(0.057)	(0.076)	(0.072)	(0.072)
CI	0.651***	0.646***	0.683***	−0.169***	−0.192***	−0.233***
	(0.044)	(0.044)	(0.046)	(0.059)	(0.056)	(0.058)
Bitter		0.079**	0.034		0.367***	0.418***
		(0.037)	(0.040)		(0.047)	(0.050)
CI*Bitter			0.210***			−0.233**
			(0.074)			(0.093)
Constant	3.903***	3.892***	3.902***	3.300***	3.247***	3.236***
	(0.155)	(0.155)	(0.153)	(0.207)	(0.195)	(0.193)
Observations	467	467	467	467	467	467
R-squared	0.326	0.333	0.344	0.037	0.153	0.164
Adj R-squared	0.317	0.323	0.333	0.025	0.140	0.149
F	37.12***	32.72***	30.07***	2.97**	11.80***	11.22***

CI denotes TCM culture identity; Bitter denotes perceived TCM bitterness; PU denotes perceived TCM usefulness; PUN denotes perceived TCM uncertainty. Standard errors are in parenthesis. *p < 0.05; **p < 0.01; ***p < 0.001.

To elucidate the boundary conditions of the dual mediation pathways, we conducted moderated mediation analysis across varying levels of TCM cultural identity (mean ±1 SD) (31). As detailed in Table 9, two critical patterns emerged: First, the perceived usefulness pathway exhibited conditional significance - the indirect effect shifted from non-significant negativity (*β* = −0.021, *p* > 0.05) at low TCM cultural identity (−1 SD) to robust positivity (*β* = 0.088, *p* < 0.001) at high TCM cultural identity (+1 SD), establishing TCM cultural identity as an amplifier of perceived TCM usefulness (H6 supported). Second, the perceived TCM uncertainty-mediated suppression effect is attenuated progressively from strong negativity (*β* = −0.085, *p* < 0.001) to less significance (*β* = −0.046, *p* < 0.01) across TCM cultural identity levels, revealing TCM cultural identity’s buffering role against perceived TCM uncertainty (H8 validated).

**Table 9:** Results of conditional indirect effects.

DV	Mediator	Moderator		Conditional indirect effect	SE	95% Confidence interval	
						Lower	Upper
TCMI	PU	CI	M-1 SD	−0.021	0.032	−0.087	0.044
			M	0.033**	0.017	0.001	0.069
			M + 1 SD	0.088***	0.023	0.048	0.139
	PUN		M-1 SD	−0.085***	0.029	−0.150	−0.034
			M	−0.066***	0.021	−0.110	−0.028
			M + 1 SD	−0.046**	0.018	−0.092	−0.018

Results are based on 5,000 bootstrap resamples. DV denotes dependent variable. M denotes Mean. SD denotes standard deviation. SE denotes standard error. CI denotes TCM culture identity; PU denotes perceived TCM usefulness; PUN denoted perceived TCM uncertainty. *p < 0.05; **p < 0.01;***p < 0.001.

## 4 Discussion

### 4.1 Key findings

On the one hand, we find that individuals who have higher levels of TCM bitterness perception would assign more usefulness perception to TCM, increasing their intention to use TCM. Scientific evidence substantiates that herbal bitterness serves as a chemosensory marker of bioactive potential in TCM theory (6, 32). Clinical TCM formulations targeting critical health conditions - spanning cancer, diabetes, stroke, heart diseases, digestive diseases - predominantly incorporate synergistic combinations of bitter botanicals (4). If people make more bitterness-therapeutic indication association, they will be more inclined to tolerate the bitterness (33). However, when contextualized within meta-analytic norms of bitterness-influenced decision-making (34), where absolute standardized effects for taste perceptions on product evaluations often range around 0.15, the observed bitterness-usefulness association (*β* = 0.079) lies at the lower bound. This reinforces the interpretation that, although statistically discernible, the direct sensory cue of bitterness is a relatively weak predictor of perceived usefulness compared to other factors such as cultural identity or prior knowledge.

On the other hand, the results also indicate that higher levels of TCM bitterness perception have negative indirect effect on intention to use TCM by arousing more perceived TCM uncertainty. Bitter taste is thought to have evolved as a deterrent against ingesting toxic substances (6) and may increase people’s uncertainty perception about the “toxicity” or “side effects” of TCM. Individuals’ innate aversion to bitter taste may generalized to overall uncertainty assessment of TCM as well. As evidence from neuroscience suggests that bitter stimulus activates the insula (35) which is also involved in risk decision-making (27). While the evolutionary rationale that bitter taste signals potential toxicity provides a foundational premise (6), its translation into heightened uncertainty in the specific context of TCM is best understood through the psychological lens of generalized risk aversion. Research on psychology maintained that bitterness triggers negative emotions (such as anxiety), which in turn interferes with rational judgment and amplifies the perception of uncertainty in decision making (36). In line with this thought, higher levels of TCM bitterness perception would be associate with more uncertainty perception to TCM, suppressing people’s intention to use TCM.

The above findings elucidate a dual-layered nature of the ‘double-edged sword’ effect. First, at the cognitive activation level, bitterness as a potent sensory stimulus shows a much stronger innate association with ‘potential risk’ (uncertainty) than its culturally learned association with ‘therapeutic efficacy’ (usefulness), as reflected in the significant difference in first-stage path coefficients. Second, at the behavioral transmission level, because the two mediating cognitions differ in their inherent strength of relationship with intention (path coefficients: 0.464 and −0.156), they are differentially amplified or attenuated during transmission. Ultimately, this leads to relatively balanced positive and negative net effects of bitterness on intention, which statistically cancel each other out, resulting in a near-zero total indirect effect and a non-significant total effect (*p* = 0.458).

The results also indicate that higher levels of TCM bitterness perception have negative indirect effect on intention to use TCM by arousing more perceived TCM uncertainty. Bitter taste is thought to have evolved as a deterrent against ingesting toxic substances (6) and may increase people’s uncertainty perception about the “toxicity” or “side effects” of TCM. Individuals’ innate aversion to bitter taste may generalized to overall uncertainty assessment of TCM as well. As evidence from neuroscience suggests that bitter stimulus activates the insula (35) which is also involved in risk decision-making (37). Similarly, research on psychology maintained that bitterness triggers negative emotions (such as anxiety), which in turn interferes with rational judgment and amplifies the perception of uncertainty in decision making (36). In line with this thought, higher levels of TCM bitterness perception would be associate with more uncertainty perception to TCM, suppressing people’s intention to use TCM.

Apart from assessing the path associations between perceived TCM bitterness and intention to use TCM proposed by the SOR paradigm, this study introduces a novel moderated mediation architecture by identifying TCM cultural identity as a critical boundary condition that systematically moderates both the mediating pathways (perceived TCM usefulness and uncertainty) and their countervailing effects. Scholars have reached a consensus that TCM culture is deeply rooted in traditional Chinese culture (10–12) whose philosophical system, thinking paradigm and value concepts will subtly influence people’s decision-making. In line with this thought, previous studies on TCM culture usually applied individual TCM cultural background (38, 39) or TCM cultural identity (40–42) as antecedent variables to predict individuals’ behavior. For example, Chinese-Americans are more inclined to choose TCM to treat mental illness given that TCM culture alleviates the shame of seeking medical treatment for mental illness (43). more than 60% of Chinese immigrants in Canada use a combination of Chinese and Western medicine to maintain their health due to the acknowledgment of TCM theory (39). In the herbal medicine innovation process, western researchers often fail to capture the tacit knowledge embedded in the ancient texts, which makes them skeptical of the science of TCM (44). In view of the subtle influence of TCM culture on people’s behavior, this study assumes that strong TCM cultural identity strengthens the ‘bitterness-usefulness’ pathway by activating and reinforcing the culturally learned heuristic ‘good medicine tastes bitter,’ making this association more accessible and credible. Additionally, strong TCM cultural identity weakens the ‘bitterness-uncertainty’ pathway by providing a pre-existing, trusted framework for interpreting bodily sensations and treatment experiences, thereby buffering the default negative affective response and reducing its generalization into broader treatment doubts.

To further assess the robustness and theoretical specificity of our proposed moderated mediation model, we tested a logical alternative wherein TCM cultural identity moderates the second-stage relationships (the paths from perceived usefulness/uncertainty to intention). The results showed that these second-stage interaction effects (CI × PU and CI × PUN) were statistically non-significant. This finding reinforces the proposed theoretical architecture: cultural identity operates primarily at the initial cognitive appraisal stage, shaping how bitterness is interpreted as a signal of either usefulness or uncertainty, rather than directly modifying how these established cognitions translate into behavioral intention. The non-significance of the alternative model strengthens the conclusion that our first-stage moderated mediation framework provides a more coherent account of the data and the underlying decision-making process.

### 4.2 Implications

First, by applying the SOR framework, we untangle the internal mechanism linking TCM bitterness perception to usage intention. Within this paradigm, bitterness perception is operationalized as the external gustatory Stimulus (S). This stimulus activates two distinct internal Organismic (O) states: the cognitive appraisal of perceived TCM usefulness and perceived TCM uncertainty. These competing organismic states, in turn, drive the final behavioral Response (R), which is the intention to use TCM. According to the SOR-based view, a relationship occurs between stimulus and response because of the activated organism (13). We address the black box of how bitterness perception is correlate with intention to use TCM by examining the mediating (suppression) roles of organisms in terms of perceived TCM usefulness (uncertainty). Particularly, the positive direct effect between TCM bitterness perception and the intention to use TCM is opposite in sign to the negative indirect effect through perceived TCM uncertainty, which means that the perceived TCM uncertainty suppresses the positive effect of TCM bitterness perception on the intention to use TCM. This can explain why people’s decision-making toward using TCM or not are neutral even they bear in mind that bitterness is a symbolic recognition of TCM efficacy. The observed pattern, where the positive direct effect is suppressed by a negative indirect effect via uncertainty, is not merely a statistical artifact but reflects a theoretically meaningful conflict. It can be interpreted through the lens of dual-process theories (45), wherein the bitter taste triggers a rapid, affectively negative, and uncertainty-inducing intuitive response (linked to innate aversion), which competes with a slower, culturally conditioned cognitive appraisal associating bitterness with efficacy. The net non-significant total effect underscores that for many individuals, the decision is characterized by an internal competition between these two systems. In real-world treatment scenarios, TCM treatment course is long, individuals often require to take medicine many times in order to verify therapeutic effects. One might speculate that the immediate perception of bitterness-uncertainty becomes amplified when uncertain treatment effect coexists with noticeable taste discomfort, overshadowing expectations for therapeutic benefits.

Moreover, this investigation extends prior research by systematically examining how TCM cultural identity moderates the relationship between perceived TCM bitterness and intentions to use TCM, thereby advancing theoretical understanding of sensory-cognitive interactions in healthcare decision-making. TCM culture embodies a philosophical system, thinking patterns, and values that serve as the basis for people to make decisions (40). An aversion to bitter taste has a robust innate component, the specific attribution of bitterness to medicinal potency is a culturally acquired cognitive schema. This is supported by cross-cultural research on taste perceptions and health beliefs (46). Thus, the bitterness-usefulness link is contingent upon exposure to and internalization of specific cultural narratives, such as the ‘good medicine tastes bitter’ proverb. The results of this study show that TCM culture identity can strengthen people’s psychological anchoring of ‘good medicine tastes bitter’, while at the same time alleviating the generalized uncertainty perception induced by bitterness perception, thus ultimately conducive to promoting people’s intention to use TCM in a two-pronged way.

The findings also offer significant insights into TCM promotion practice. First, the practical implications of the bitterness-usefulness pathway must be interpreted with caution due to its small effect size. Promotional strategies aiming solely to reinforce the ‘bitterness signifies potency’ association are unlikely to yield substantial shifts in public intention to use TCM, given the minimal variance explained. Instead, practitioners and policymakers should prioritize addressing the stronger negative pathway (bitterness-uncertainty) and leveraging the potent moderating role of cultural identity, which demonstrated larger and more robust effects in our model. Second, the actors in promotion of TCM should scientifically understand the double-edged sword effect mechanism of the bitterness perception of TCM on people’s intention to use TCM. It is necessary to conduct pharmacological validation of bitter compounds and implement a “bitter-efficacy quotient” labeling system (quantifying therapeutic potency per bitterness unit) to enhance consumer confidence in TCM’s therapeutic value. At the same time, it is also necessary to establish rigorous toxicological profiling protocols to develop international-standard safety evaluation frameworks with clearly defined safety thresholds, thereby addressing public concerns regarding herbal medicine safety. Finally, the deep power of TCM culture lies in transforming physiological stimulation into cultural identity, thereby shaping a unique drug cognition, so that the intention to use TCM goes beyond simple sensory judgment and sublimates into cultural consciousness.

### 4.3 Limitations and future research

While providing novel insights, this study presents several limitations that might be served as agenda for future research. First and foremost, the primary limitation of this study lies in its sampling bias. The participants were drawn from a medical university, representing a population with significantly higher TCM literacy than the general public. As noted by the reviewer, this group represents a specialized segment (<1% of the population) possessing the cognitive framework to decode bitterness as efficacy. Consequently, the observed positive link between bitterness and perceived usefulness (*β* = 0.079) likely represents a best-case scenario. To further evaluate the reliability of this statistically significant pathway, we performed a post-hoc power analysis using G*Power (3.1.9.7) under the “R^2^ increase” framework (47). With *α* = 0.05, sample size *n* = 467, total number of predictors *k* = 6, the number of tested predictors *k* = 1 (the core predictor, bitter taste perception), and *f*^2^ = 0.013, the analysis yielded a statistical power of 70%—below the widely adopted 80% benchmark. Thus, while the bitterness–usefulness path reached statistical significance, its small effect size coupled with suboptimal power warrants caution in interpreting the stability and practical relevance of this relationship. In a general, TCM-naïve population, this positive pathway might be non-existent, leaving only the negative uncertainty pathway. Therefore, caution must be exercised when generalizing these findings to the broader public. Thus, replication through random sampling in alternative populations, such as people without medical background is essential for future research.

Second, a critical limitation regards the measurement validity of bitterness perception. This study relied on retrospective self-reports containing affective items (e.g., ‘makes me feel disgusting’), which conflates physiological sensory intensity with emotional reaction and post-consumption rationalization. Consequently, the variable bitterness perception in our model reflects a subjective, compound psychological experience rather than an objective psychophysical measure of taste intensity. This distinction is crucial, as the reported bitterness is already filtered through the individual’s emotional bias. To address the limitations of retrospective survey measures, future research requires rigorous psychophysical validation. We recommend employing controlled taste tests using standardized bitter stimuli (e.g., quinine solutions) and Labeled Magnitude Scales (LMS) to capture sensory intensity separately from hedonic response. This would allow researchers to disentangle whether the ‘double-edged sword’ effect is driven by the actual intensity of the taste or the affective aversion it provokes.

Thirdly, our sample exhibited an exceptionally high level of TCM Cultural Identity (Mean = 4.458 on a 5-point scale), which significantly deviates from the likely distribution in the general population (41). This ceiling effect in cultural identity serves as a boundary condition for our model. It suggests that the bitterness-efficacy heuristic is heavily dependent on prior cultural indoctrination. Future research should replicate this model in community samples with lower median cultural identity to verify if the positive mediation path collapses absent this cultural buffer.

Fourthly, the cross-sectional data cannot rule out several alternative explanations for the observed associations. The reported paths, while theoretically derived and tested within an established framework, indicate significant associations rather than confirmed causal effects. The relationship between bitterness and perceived usefulness of TCM can be strengthened through alternative interpretations. While commonsense suggests that individuals make choices based on pre-existing preferences, extensive social psychology literature indicates that choices can, in fact, shape preferences. Preferences at least partially guide choices, but this relationship is bidirectional: choices themselves can feedback and modify preferences (48). According to cognitive dissonance theory, when individuals make a choice between two options, they reduce dissonance by adjusting their evaluation of the selected option, ultimately forming new preferences. For individuals who appreciate TCM culture and understand its core principles, bitterness can be rationalized and redefined as a salient indicator of therapeutic efficacy, constituting an alternative cognitive mechanism underlying bitterness perception (49). In families with a longstanding history of TCM use, a shared cultural identification with TCM—encompassing collective acceptance of its core principles and practices—gradually develops among family members (50). This shared cultural background provides multi-level cognitive support when individuals encounter specific experiences, such as the bitterness of TCM herbs.

At last, this study examines respondents’ intention to use TCM by self-reporting behavior intention. There may be differences between actual behavior and behavioral intention. Future research can provide more substantial evidence by follow-up questionnaire about the actual TCM using behavior.

## 5 Conclusion

This study reveals double-edged effects that TCM bitterness perception simultaneously activates competing cognitive evaluations—enhancing perceived TCM usefulness while amplifying perceived TCM uncertainty. These opposing pathways create countervailing indirect effects on intention to use TCM, with perceived TCM usefulness exerting a positive motivational force and perceived TCM uncertainty generating behavioral hesitation. Crucially, TCM cultural identity emerges as a pivotal moderating variable that differentially moderating these mediation channels: it strengthens the bitterness-usefulness association while attenuating the bitterness-uncertainty linkage, thereby resolving the paradox through cultural-cognitive filtering. The adapted SOR framework demonstrates robust explanatory power in modeling this sensory-behavioral duality, positioning bitterness perception not merely as a phytochemical signal but as a culturally embedded decision heuristic that interacts with medical epistemology to shape therapeutic choices.

## Data Availability

The raw data supporting the conclusions of this article will be made available by the authors, without undue reservation.
